# Trends in the development process of clinical practice guidelines: a questionnaire survey for the guideline development groups in Japan

**DOI:** 10.1186/s12913-022-07492-7

**Published:** 2022-01-21

**Authors:** Yosuke Hatakeyama, Kanako Seto, Koki Hirata, Ryo Onishi, Kunichika Matsumoto, Tomonori Hasegawa

**Affiliations:** Department of Social Medicine, Toho University School of Medicine, 5-21-16, Omori-Nishi, Ota-ku, Tokyo, 143-8540 Japan

**Keywords:** Clinical practice guidelines, Guideline development, Questionnaire

## Abstract

**Background:**

Clinical practice guidelines (CPGs) are representative methods for promoting healthcare standardization and improving its quality. Previous studies on the CPG (published by 2006) development process in Japan reported that the involvement of experts and patients, efficient evidence collection and appraisal, and paucity of evidence on Japanese patients should be improved for the efficient CPG development. This study aimed to clarify the trends of CPG development process in Japan, focusing on the involvement of experts and patients, efficient evidence collection and appraisal, and paucity of Japanese evidence.

**Methods:**

A cross-sectional questionnaire survey was conducted for CPG development groups to collect information on the development activities of the CPGs published from 2012 to 2019. These CPGs were identified from the Japanese guideline clearinghouse. The questionnaire included the questions on composing the group, securing funding sources, collecting and appraising the research evidence, and the difficulties in the CPG development process. The questionnaires were distributed to the chairpersons of the CPG development groups through postal mail from November 2020 to January 2021. Combining the data from the current survey with those of previous studies reporting the development process of CPGs published by 2011, we analyzed the trend in the CPG development process.

**Results:**

Of the total 265 CPGs included in the analysis, 164 (response rate: 41.4%) were from the current survey and 101 (response rate: 44.5%) were from previous studies. Among these, 40 (15.1%) were published by 2005, 47 (17.7%) in 2006–2010, 77 (29.1%) in 2011–2015, and 101 (38.1%) in 2016–2019. The proportion of CPGs involving methodologists did not increase through the publication periods. The proportion of CPGs involving patients almost doubled from the first period (15.9%) to the fourth period (32.4%). The yield rates of the articles did not change through the publication periods. The difficulty in “Coping with the paucity of Japanese evidence” has been improving consistently (69.2% in the first period to 37.4% in the fourth period).

**Conclusions:**

Our results suggest the need for methodological improvement in the efficient collection and appraisal of evidence and in the system assigning experts to the CPG development groups.

**Supplementary Information:**

The online version contains supplementary material available at 10.1186/s12913-022-07492-7.

## Background

Clinical practice guidelines (CPGs) are statements that include recommendations based on a systematic review of evidence and an assessment of the benefits and harms of alternative care options in order to assist the decision making of practitioners and patients [1, 2]. CPGs are representative methods for promoting the standardization of healthcare and improving its quality. In October 2021, more than 29,000 articles indexed as “practice guideline” for publication type were listed in PubMed. In Japan, the Ministry of Health, Labour and Welfare has encouraged academic societies to develop CPG for major diseases using public research funds since 2000. Currently, academic societies and research groups are involved in developing and managing CPGs, and approximately 60 CPGs, including newly developed and revised CPGs, are being published every year.

Numerous development manuals and more than 40 appraisal tools have been published to ensure the quality of CPGs [3, 4]. The general steps involved in the development of CPG are as follows: i) identifying and refining the CPG subject area, ii) running development groups, iii) identifying and assessing the research evidence, iv) translating the evidence into CPGs, and v) reviewing and updating the CPGs [5]. Because the evidence and resources that can be used differ among the CPG developers, the actual CPG development processes may vary substantially [2]. Therefore, information regarding the methods used and the difficulties encountered in the actual development processes could help in improving the environment (available methodological guidance, tools, support systems, etc.) of CPG development process. Additionally, given the changing environments of CPG development, the trends of the CPG development process can provide valuable information. The descriptions of the methods and processes of CPG development are usually included in the CPGs, and there are some case reports [6, 7] and cross-sectional surveys [8–10] on the actual CPG development processes. These reports show only a snapshot of the development process and not the trends of the methods used by CPG developers. Some previous studies have reported the trends of the CPG development through conducting systematic reviews [11–13]. However, it is not possible to obtain information on the detail of ingenuity or difficulties in the CPG development process from the description in published CPGs only.

Based on the questionnaire surveys conducted on the development groups of the CPGs published by 2006 [14–16], Hasegawa revealed the problems in the CPG development process in Japan. These problems were associated with the involvement of experts (e.g., epidemiologists, librarians, or health economists) and patients, efficient evidence collection and appraisal in systematic review, and paucity of evidence on Japanese patients [16]. The US Institute of Medicine pointed out the importance of expert and patient involvement in the “Guideline Development Group Composition” for developing trustworthy CPGs [2]. Participation of patients as stakeholders in the CPG development process is expected to enhance the validity and usefulness of published CPGs [17]. Because conducting systematic reviews can be a time-consuming and cost- and resource-intensive task, the efficacy of systematic reviews becomes especially problematic when the CPG developers make recommendations expeditiously [18]. CPGs are developed to support patients and practitioners in each country or region based on the evidence gathered from around the world, and the evidence used in the CPGs may often not include patients of interest of the CPG developers. These problems were revealed on examination of the CPG development processes in Japan, but these could be common problems for CPG developers worldwide. Hasegawa et al. conducted a questionnaire survey on development groups of the CPG published by 2011, addressing the CPG development process. Their questionnaire and data can be used in this study for the trend analysis of the methods of CPG development and the difficulties encountered by the CPG development groups [19].

## Methods

### Aim

This study aimed to clarify the trends of CPGs development process in Japan, focusing on the involvement of experts and patients, efficient evidence collection and appraisal in systematic review, and paucity of evidence on Japanese patients.

### Study design and participants

A cross-sectional questionnaire survey conducted for Japanese CPG development groups was used to collect information on the development activities of CPGs published from 2012 to 2019.

To identify Japanese CPGs, we used a Japanese guideline clearinghouse managed by the Toho University Medical Media Center [20], which collected all CPGs published in Japan. The CPGs were selected based on the following criteria: (1) the title includes the terms “guideline,” “guidance,” or “guide”; (2) the methodology describes the CPG development process based on existing evidence or newly conducted systematic reviews; and (3) the theme relates to clinical practice and not to topics such as medical ethics and animal experimentation. The CPGs whose target readers were patients were excluded from this study.

The survey was conducted through postal mail, targeting chairpersons of the CPGs identified from the Japanese guideline clearinghouse, from November 2020 to January 2021. The bibliographic information about the CPG for which we requested to answer were enclosed with the questionnaire to minimize the potential discrepancies between CPGs participants answered and those we intended. We also sent reminders in December 2020 to the unresponsive chairpersons. We retrieved information on the chairpersons from the descriptions in the CPGs. To ensure continuity in the analysis, the questionnaire was similar to that of the previous studies regarding the questions on a) the processes for composing the development group (total number of members and the experts and patients involved), and collecting and appraising the research evidence, and b) the difficulties in the CPG development processes [14–16, 19]. The terminology of these items was based on a Japanese guidebook for CPG development [21] and the translated Japanese version of the original Appraisal of Guidelines for Research and Evaluation (AGREE) instrument [22, 23].

The Ethics Committee of Toho University School of Medicine stated that this study was not applicable for ethical review under the Japanese regulations (No. A20064). All participants were informed about the objective of the research and the policy for keeping their data confidential and anonymous in the survey. There is no published protocol of this study. For the reporting, we followed the Strengthening the Reporting of Observational Studies in Epidemiology (STROBE) guideline for observational studies [24].

### Measurements

Information about the publication year, development group, and versions of the CPGs were collected from the descriptions in the CPGs. The publication years of the CPG were divided into four periods: by 2005, from 2006 to 2010, from 2011 to 2015, and from 2016 to 2019. Development groups were grouped into three categories: research group, research group plus academic society, and academic society. “Research groups” were temporary groups for conducting research on specific themes. In Japan, a CPG development handbook explaining the methodology related to MINDS, a business arm associated with the CPGs of the Japan Council for Quality Care, has served as the basis for the development of CPGs for a considerable time [7, 25]. Furthermore, it encouraged the CPG developers to conduct an evidence appraisal using abstract forms consisting of bibliographic information, structured summaries, and comments from abstractors. Therefore, evidence searching, evidence appraising with abstract forms, and evidence citing in completed CPGs were set as the process of evidence collection and appraisal in the questionnaire. The yield rates were calculated by dividing the number of appraised articles by the number of obtained articles and the number of cited articles by the number of obtained articles through the search.

### Data analysis

We used data of the CPGs published in 2012–2019 from the current survey and those published by 2011 from previous studies [14–16, 19]. To confirm whether the challenges of CPG development suggested in the previous study [16] have been resolved, we analyzed the data using the Mantel-Haenszel test for trend for categorical variables and the Jonckheere-Terpstra test for continuous variables. All data were analyzed using Statistical Product and Service Solutions software, version 25.0 (IBM), and *P*-values < 0.05 were considered statistically significant.

## Results

### CPG selection

**Fig. 1:**
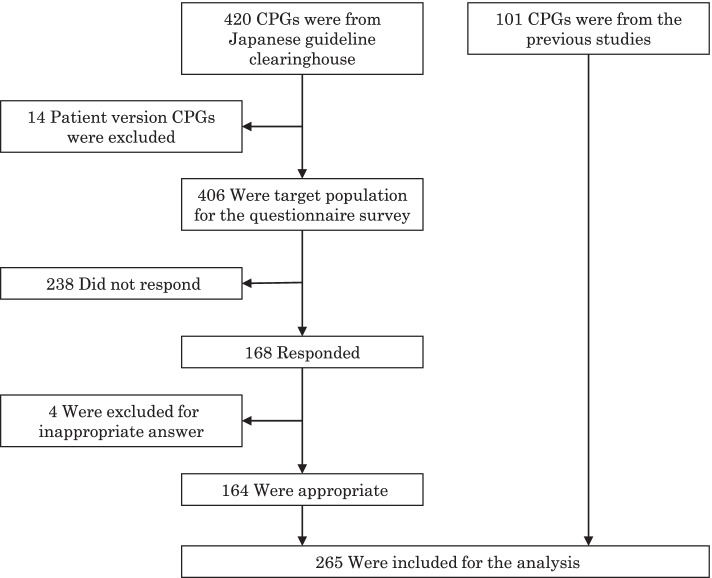
Flowchart of the selection of 265 CPGs from Japanese guideline clearinghouse and previous studies. We included data of the CPGs published in 2012–2019 from the current survey and those published in 2011 from previous studies [14–16, 19]. Of the 168 CPGs, 4 were excluded because the respondents answered for versions of CPGs different from those expected by us. Of the total 265 CPGs, 164 were from the current survey and 101 were from previous studies [14–16, 19]. Abbreviations: CPG, clinical practice guideline

Figure 1 shows the flowchart of the selection of CPGs in this study. In this study, 420 CPGs were retrieved from the Japanese guideline clearinghouse, of which 14 CPGs for patients were excluded. The questionnaires were distributed to the chairpersons of 406 CPG development groups. The response rate was 41.4% (168/406), and four CPGs were excluded because the chairpersons of these CPG development groups answered for versions of CPG different from those expected by us. The response rate of previous studies was 44.5% (101/227). Finally, 265 CPGs were included in the analysis, of which 164 (61.9%) were from the Japanese guideline clearinghouse and 101 (38.1%) were from previous studies [14–16, 19].

The characteristics of the 265 included CPGs are listed in Table 1. Among these, 40 (15.1%) were published by 2005, 47 (17.7%) in 2006–2010, 77 (29.1%) in 2011–2015, and 101 (38.1%) in 2016–2019. The proportion of CPG developed by academic societies has increased from 45.0% in the first period (by 2005) to 86.1% in the fourth period (2016–2019).

**Table 1:** Characteristics of the CPGs included in this study (*N* = 265)

		Publication period				*P* ^a^
		by 2005	2006–2010	2011–2015	2016–2019	
		(*N* = 40)	(*N* = 47)	(*N* = 77)	(*N* = 101)	
Development group						
Research group	n (%)	18 (45.0)	2 (4.3)	13 (16.9)	10 (9.9)	< 0.001
Research group + academic society	n (%)	4 (10.0)	0 (0.0)	3 (3.9)	4 (4.0)	
Academic society	n (%)	18 (45.0)	45 (95.7)	61 (79.2)	87 (86.1)	
Rate of public funding in CPG development cost						
< 50%	n (%)	17 (50.0)	35 (81.4)	45 (77.6)	57 (72.2)	0.124
> 50%	n (%)	17 (50.0)	8 (18.6)	13 (22.4)	22 (27.8)	
Version						
First	n (%)	32 (80.0)	32 (68.1)	46 (59.7)	46 (45.5)	< 0.001
Revised	n (%)	8 (20.0)	15 (31.9)	31 (40.3)	55 (54.5)	

^a^ Mantel-Haenszel test for trend

Abbreviations: *CPG* clinical practice guideline

### Expert and patient involvement in the guideline development groups

Table 2 shows the results regarding the group members and collaborations of the CPG development process in each publication period. The total number of members involved in CPG development has been increasing. The mean (standard deviation: SD) number of members in the first, second, third, and fourth publication period was 25.5 (14.4), 23.9 (18.2), 26.5 (30.3), and 37.2 (36.2), respectively. The mean (range) numbers were 24.3 (0 to 183) in Specialists for the theme of CPGs, 3.0 (0 to 61) in General practitioners engaged in medical practice of the theme of CPGs, and 1.1 (0 to 23) in Comedies, respectively. The proportion of CPGs involving methodologists (epidemiologists, statisticians, evidence-based medicine experts, experts in the guideline development, or librarians) and health economists did not increase through the publication periods. The proportion of CPGs involving patients almost doubled from the first period (12.5%) to the fourth period (32.4%). Additionally, collaboration between the CPG development groups and patient groups showed an increasing trend from 5.4% in the first period to 21.2% in the fourth period.

**Table 2:** Total number of members and collaborations with other organizations (*N* = 265)

		Publication period				*P*
		by 2005	2006–2010	2011–2015	2016–2019	
		(*N* = 40)	(*N* = 47)	(*N* = 77)	(*N* = 101)	
Number of members	mean (SD)	25.5 (14.4)	23.9 (18.2)	26.5 (30.3)	37.2 (36.2)	0.036 ^b^
CPGs including						
Methodologist ^a^	n (%)	20 (52.6)	26 (55.3)	34 (48.6)	57 (64.0)	0.228 ^c^
Health economist	n (%)	8 (20.0)	7 (14.9)	12 (15.6)	17 (16.8)	0.796 ^c^
Patient representative	n (%)	5 (12.5)	4 (8.7)	12 (15.6)	33 (32.4)	0.001 ^c^
CPGs collaborating with						
Academic society	n (%)	29 (78.4)	36 (76.6)	60 (77.9)	79 (79.0)	0.842 ^c^
Medical association	n (%)	8 (21.6)	10 (22.7)	12 (15.8)	18 (18.4)	0.524 ^c^
Patient group	n (%)	2 (5.4)	5 (11.1)	10 (13.5)	21 (21.2)	0.014 ^c^

^a^ "Methodologist" includes epidemiologists, statisticians, evidence-based medicine experts, experts in the guideline development, or librarians

^b^ Jonckheere-Terpstra test

^c^ Mantel-Haenszel test for trend

Abbreviations: *CPG* clinical practice guideline, *SD* standard deviation

### Evidence collection and appraisal

As shown in Table 3, the activities for evidence collection and appraisal changed slightly. Regarding the databases for searching evidence, PubMed was the most used database by more than 90% of the CPG development groups, followed by Ichushi, a Japanese database managed by the Japan Medical Abstracts Society, which was used by about 80% of the groups in all the publication periods. The proportion of the CPG development groups using JMEDplus in the evidence search, another database listing Japanese articles, showed a decreasing trend. There were no trends observed for the number of articles searched, appraised, and cited in the CPG. Additionally, the yield rates of the articles did not show any improvement trends. While the proportion of the CPG involving two or more reviewers for citing articles showed a consistently increasing trend (from 43.3% in the first period [by 2005] to 78.7% in the fourth period [2016–2019]), those for searching and appraising articles showed no observable trend.

**Table 3:** Evidence collection and appraisal process (*N* = 265)

			Publication period				*P*
			by 2005	2006–2010	2011–2015	2016–2019	
			(*N* = 40)	(*N* = 47)	(*N* = 77)	(*N* = 101)	
Database							
Ichushi ^a^		n (%)	29 (87.9)	35 (78.3)	64 (83.1)	84 (82.2)	0.762 ^b^
JMEDplus ^a^		n (%)	9 (27.3)	12 (26.1)	10 (13.0)	8 (7.9)	0.001 ^b^
PubMed		n (%)	37 (92.5)	46 (100.0)	74 (96.1)	101 (99.0)	0.123 ^b^
Cochrane		n (%)	21 (52.5)	25 (56.5)	48 (62.3)	61 (60.4)	0.366 ^b^
Number of articles							
Searched		mean (SD)	7441.7 (13,052.7)	3721.0 (6460.0)	3924.4 (5427.8)	5772.4 (10,802.2)	0.826 ^c^
Appraised		mean (SD)	603.5 (708.0)	303.9 (304.4)	638.6 (1050.0)	550.7 (923.9)	0.823 ^c^
Cited		mean (SD)	305.6 (208.7)	377.5 (349.9)	493.6 (783.1)	350.9 (329.4)	0.806 ^c^
Yield rate of articles							
Appraised/Searched		mean (SD)	0.155 (0.219)	0.267 (0.268)	0.315 (0.356)	0.241 (0.270)	0.402 ^c^
Cited/Searched		mean (SD)	0.165 (0.241)	0.312 (0.285)	0.346 (0.337)	0.182 (0.201)	0.841 ^c^
CPGs using two or more reviewers for							
Searching		n (%)	16 (60.0)	15 (55.6)	15 (45.5)	48 (77.4)	0.059 ^b^
Appraising		n (%)	15 (78.9)	16 (80.0)	16 (61.5)	45 (78.9)	0.963 ^b^
Citing		n (%)	13 (43.3)	18 (54.5)	24 (61.5)	48 (78.7)	0.001 ^b^

^a^ Ichushi and JMEDplus are databases for Japanese medical journal articles

^b^ Mantel-Haenszel test for trend

^c^ Jonckheere-Terpstra test

Abbreviations: *CPG* clinical practice guideline, *SD* standard deviation

### Difficulties in the guideline development process

Table 4 presents the difficulties encountered in the CPG development process. The highest difficulty was observed in the process of “handling with parts without evidence” (65.7%), followed by “evaluating the evidence” (60.6%), “editing” (51.5%), “searching for the research evidence” (47.5%), “composing development group” (45.5%), and “coping with the paucity of Japanese evidence” (37.4%) in the fourth publication period. “Coping with the paucity of Japanese evidence” has been improving consistently. Difficulty in the process of “composing development group” increased from the first publication period (by 2005) to the fourth (2016–2019). While there were no observable trends in the number and yield rate of the articles (Table 3), the CPG development groups showed an increasing trend of having difficulty in the process of “searching for the research evidence”.

**Table 4:** Difficulties in the clinical practice guidelines development process (*N* = 265)

		Publication period				*P* ^a^
		by 2005	2006–2010	2011–2015	2016–2019	
		(*N* = 40)	(*N* = 47)	(*N* = 77)	(*N* = 101)	
Composing development group	n (%)	10 (25.6)	9 (20.5)	33 (42.9)	45 (45.5)	0.003
Searching for the research evidence	n (%)	9 (23.1)	8 (20.5)	36 (46.8)	47 (47.5)	0.001
Evaluating the evidence	n (%)	21 (53.8)	22 (50.0)	45 (58.4)	60 (60.6)	0.285
Handling with parts without evidence	n (%)	20 (51.3)	27 (61.4)	53 (68.8)	65 (65.7)	0.128
Coping with the paucity of Japanese evidence	n (%)	27 (69.2)	30 (68.2)	40 (51.9)	37 (37.4)	< 0.001
Editing	n (%)	20 (51.3)	22 (50.0)	36 (46.8)	51 (51.5)	0.958
Coordinating with other organizations	n (%)	6 (15.4)	4 (9.1)	10 (13.0)	19 (19.2)	0.295
Resolving misunderstanding on standardization and EBM	n (%)	12 (30.8)	9 (20.9)	9 (11.7)	20 (20.2)	0.209
Securing funds	n (%)	8 (20.5)	6 (13.6)	13 (16.9)	19 (19.2)	0.869
Getting support from CPG experts	n (%)	5 (12.8)	0 (0.0)	15 (19.5)	14 (14.1)	0.229

^a^ Mantel-Haenszel test for trend

Abbreviations: *CPG* clinical practice guideline, *EBM* evidence-based medicine

## Discussion

This survey involving CPG development groups in Japan revealed that among the problems noted in CPG development in a previous study [16], there has been significant improvement in patient involvement and coping with the paucity of Japanese evidence, but not in expert involvement, efficiency of evidence collection and appraisal.

Patient involvement has increased across many fields in Japan, such as clinical practice, health policy making, and clinical research [26–28]. With respect to CPG, the Japanese CPG development handbook published in 2007 [25] served as the basis for CPG development in Japan for a considerable time [7]. It suggested that patient involvement was “desirable” in the CPG development process, but the manual, which was the updated version of that handbook in 2017, emphasized the importance of patient involvement by calling it “essential” [29]. The efforts in the dissemination of the importance of patient involvement might have enhance the validity and usefulness of the CPGs through improving patient involvement in the CPG development process.

Basing on the Grading of Recommendations Assessment, Development and Evaluation (GRADE) approach [30], the manual disseminated the assessment methods of “indirectness,” referring to the difference between the population, interventions, comparisons, and outcomes intended by the CPG developers and those in the obtained evidence [29]. Even if there was insufficient evidence of Japanese patients, the CPG developers could evaluate the body of evidence using the criteria for the certainty of evidence, including indirectness, and formulate recommendations based on such evidence. The progress in the CPG development methodology might have contributed to coping with no Japanese evidence in the CPG development process.

The CPG development methodologies, such as the GRADE approach that was developed by some of the original founders of the evidence-based medicine (EBM) movement, have improved over the last decade or more. Introducing the GRADE approach, the Japanese CPG development manual has emphasized on the comprehensiveness, transparency, and unbiasedness rather than the efficiency of systematic reviews in the CPG development process [29]. However, these methodologies may be highly technical and beyond many organized CPG efforts, including some who have formally endorsed the use of these methodologies [31]. No improvement was observed in the efficiency of evidence collection and appraisal in the results. Recently, rapid reviews, which accelerated the process of a traditional systematic review through streamlining or omitting various methods, have been conducted to produce evidence for stakeholders in a resource-efficient manner [32]. Cochrane, a global leader in the production of high-quality systematic reviews and methodological guidance, provided methodological recommendations for conducting rapid reviews [33]. Additionally, some tools for efficient systematic review have been developed and available [34, 35]. Although many challenges to the conduct of rapid reviews were addressed [36], the authors of CPG development manuals should consider introducing the methodology of rapid review and these tools to CPG developers in addition to rigorous methodologies.

With regard to expert involvement, some organizations offer support to the CPG development groups. In Japan, The MINDS Guideline Library assigns experts of CPG development to these groups [37], and several organizations, including the Japan Medical Library Association, offer support in searching for evidence in the CPG development process [38, 39]. Our results suggest that these forms of support might not fully meet the needs of the CPG development groups. Collaborating with McMaster University, Guidelines International Network (GIN), which is a global network of guideline producing organizations and guideline participants, has initiated a comprehensive, evidence-based, and up-to-date training program for CPG development group members [40]. In this program, GIN prepares the course for expert methodologist. It could increase the importance of and promote the standardization of experts. The organizations for support in the CPG development should introduce the CPG development groups about the importance of expert participation and establish an expert referral system that meets the CPG developers’ needs.

In addition to the problems noted in the previous study [16], “composing development group” has become a difficult process in CPG development. This survey revealed an increasing trend in the total number of CPG development group members and patients involved. Regarding the number of CPG development group members, Murphy et al. suggested that although having more group members increased the reliability of the group judgment, it caused coordination problems within the group [41]. As for patient involvement, Blackwood et al. recently revealed the paucity of knowledge on how to identify, incorporate and report patient preferences in CPGs through an international cross-sectional survey of CPG development organizations [42]. The lack of relevant knowledge, inability to separate personal experiences from systematic methods and analytical rules, and misunderstandings about EBM in patients makes it difficult to find an appropriate person who can consider the evidence objectively and make recommendations free of preconceived views or self-interests [2]. The increasing number of members and the progress of patient involvement in the CPG development groups might have increased the difficulty of group composition for CPG developers in the coordination and assignment of patient representatives. Piggott et al. suggested the contribution and participation of CPG development group members has become more demanding, although more guidance for CPG development is available. Therefore, they developed a guide for CPG development group members containing 33 items for consideration before, during and in follow-up to CPG group meetings and a description of each participant role [43]. The researchers on CPGs could help CPG development groups through clarifying the appropriate group composition based on the actual CPG development processes. Additionally, the support organization for the CPG development and dissemination should introduce the findings on the effective group composition including the above tool and description to CPG development groups.

This is the first study to reveal the long-term trends in CPG development activities through a cross-sectional questionnaire survey. However, it has several limitations. The response rate was not high, and there was a time lag between the CPG development and the current survey, as well as recall, selection, and unresponsive bias. The results might be biased towards the responses of the CPG development groups which have been involved in the CPG development and dissemination actively. Therefore, the results should be not interpreted as the trends for the general CPG development organizations.

## Conclusions

Although there has been improvement in the patient involvement and coping with the paucity of Japanese evidence in the CPG development process in Japan, expert involvement and efficiency of evidence collection and appraisal have not. Our results revealed the need for methodological improvement in the efficient collection and appraisal of evidence and in the supporting system assigning experts to the CPG development groups.

## Supplementary Information

**Additional file 1.**

## Data Availability

Summary data generated or analyzed during this study were included in this manuscript. If an external researcher contacts the corresponding author, the research team members will submit reviews of external provision of data to the Ethics Committee on behalf of external researchers. For data usage applications, the Ethics Committee of Toho University will examine whether the data requester can handle the data appropriately before sharing the data.

## Abbreviations

AGREE: Appraisal of Guidelines for Research and Evaluation
CPG: Clinical practice guideline
EBM: Evidence-based medicine
GIN: Guidelines International Network
GRADE: Grading of Recommendations Assessment, Development and Evaluation
STROBE: Strengthening the Reporting of Observational Studies in Epidemiology
