# Supplementary material for: Trends in the development process of clinical practice guidelines: a questionnaire survey for the guideline development groups in Japan
Source: BMC Health Serv Res. 2022 Jan 21;22:94. doi: 10.1186/s12913-022-07492-7 (PMC8783421; doi:10.1186/s12913-022-07492-7)
Supplement: Supplementary file 1 — Additional file 1. [file 12913_2022_7492_MOESM1_ESM.docx]

**Items used in the analysis extracted from the questionnaire**

**Question items for Table 2**

**Please fill in the table below regarding the numbers of guideline development member.**

| 1) Total | |  |
| --- | --- | --- |
| Background | 2) Specialists for the theme of CPGs |  |
|  | 3) General practitioners engaged in medical practice of the theme of CPGs excluding 2) |  |
|  | 4) Comedies |  |
|  | 5) Experts in clinical epidemiology or biological statistics |  |
|  | 6) Experts in the CPGs development |  |
|  | 7) Medical librarians or experts in the literature search |  |
|  | 8) Experts in health economics |  |
|  | 9) Representatives of patients |  |
|  | 10) Other |  |

**Regarding to the collaboration with related organizations**

**Please select an answer from options to each.**

**1) Other rerated academic society**

1. Collaborated from the beginning of CPG development

2. Consulted from the stage of draft completion

3. Consulted after the publication of CPG

4. Other ( )

5. No activities for collaboration

**2) Organization representing doctors (e.g. Medical association)**

1. Collaborated from the beginning of CPG development

2. Consulted from the stage of draft completion

3. Consulted after the publication of CPG

4. Other ( )

5. No activities for collaboration

**3) Patient representative organizations**

1. Collaborated from the beginning of CPG development

2. Consulted from the stage of draft completion

3. Consulted after the publication of CPG

4. Other ( )

5. No activities for collaboration

**4) Other organizations**

1. Collaborated from the beginning of CPG development

2. Consulted from the stage of draft completion

3. Consulted after the publication of CPG

4. Other ( )

5. No activities for collaboration

**Question items for Table 3**

**On the evaluation of evidence and the assessment in the body of evidence.**

**Which database did you use? Please select all applicable answer.**

a) Ichushi

b) JMEDplus

c) MEDLINE / PubMed

d) EMBASE

e) Cochrane Library

f) Other ( )

**Please tell the numbers of articles and reviewers in collecting and appraising process.**

|  | Articles | Reviewers |
| --- | --- | --- |
| 1) Searching |  |  |
| 2) Appraising with evaluating sheets (structural abstract forms) |  |  |
| 3) Citing in completed clinical practice guidelines |  |  |

**Question items for Table 4**

**Did you have the difficulties in following process? Please select all applicable answer.**

a) Composing development group

b) Searching for the research evidence

c) Evaluating the evidence

d) Handling with parts without evidence

e) Coping with the paucity of Japanese evidence

f) Editing

g) Coordinating with other organizations

h) Resolving misunderstanding on standardization and EBM

i) Securing funds

j) Getting support from CPG experts

k) Other ( )
